# The right time for senescence

**DOI:** 10.7554/eLife.72449

**Published:** 2021-11-10

**Authors:** Diogo Paramos-de-Carvalho, Antonio Jacinto, Leonor Saúde

**Affiliations:** 1 Instituto de Medicina Molecular – João Lobo Antunes, Faculdade de Medicina da Universidade de Lisboa Lisbon Portugal; 2 CEDOC, NOVA Medical School, Faculdade de Ciências Médicas da Universidade Nova de Lisboa Lisbon Portugal; 3 Instituto de Medicina Molecular – João Lobo Antunes e Instituto de Histologia e Biologia do Desenvolvimento, Faculdade de Medicina da Universidade de Lisboa Lisbon Portugal; California Institute of Technology United States; California Institute of Technology United States

**Keywords:** senescence, time, tissue remodelling, pathophysiology, senotherapies

## Abstract

Cellular senescence is a highly complex and programmed cellular state with diverse and, at times, conflicting physiological and pathological roles across the lifespan of an organism. Initially considered a cell culture artifact, senescence evolved from an age-related circumstance to an intricate cellular defense mechanism in response to stress, implicated in a wide spectrum of biological processes like tissue remodelling, injury and cancer. The development of new tools to study senescence in vivo paved the way to uncover its functional roles in various frameworks, which are sometimes hard to reconcile. Here, we review the functional impact of senescent cells on different organismal contexts. We provide updated insights on the role of senescent cells in tissue repair and regeneration, in which they essentially modulate the levels of fibrosis and inflammation, discussing how “time” seems to be the key maestro of their effects. Finally, we overview the current clinical research landscape to target senescent cells and contemplate its repercussions on this fast-evolving field.

## Cellular senescence: a story between life and death

**Figure 1.**
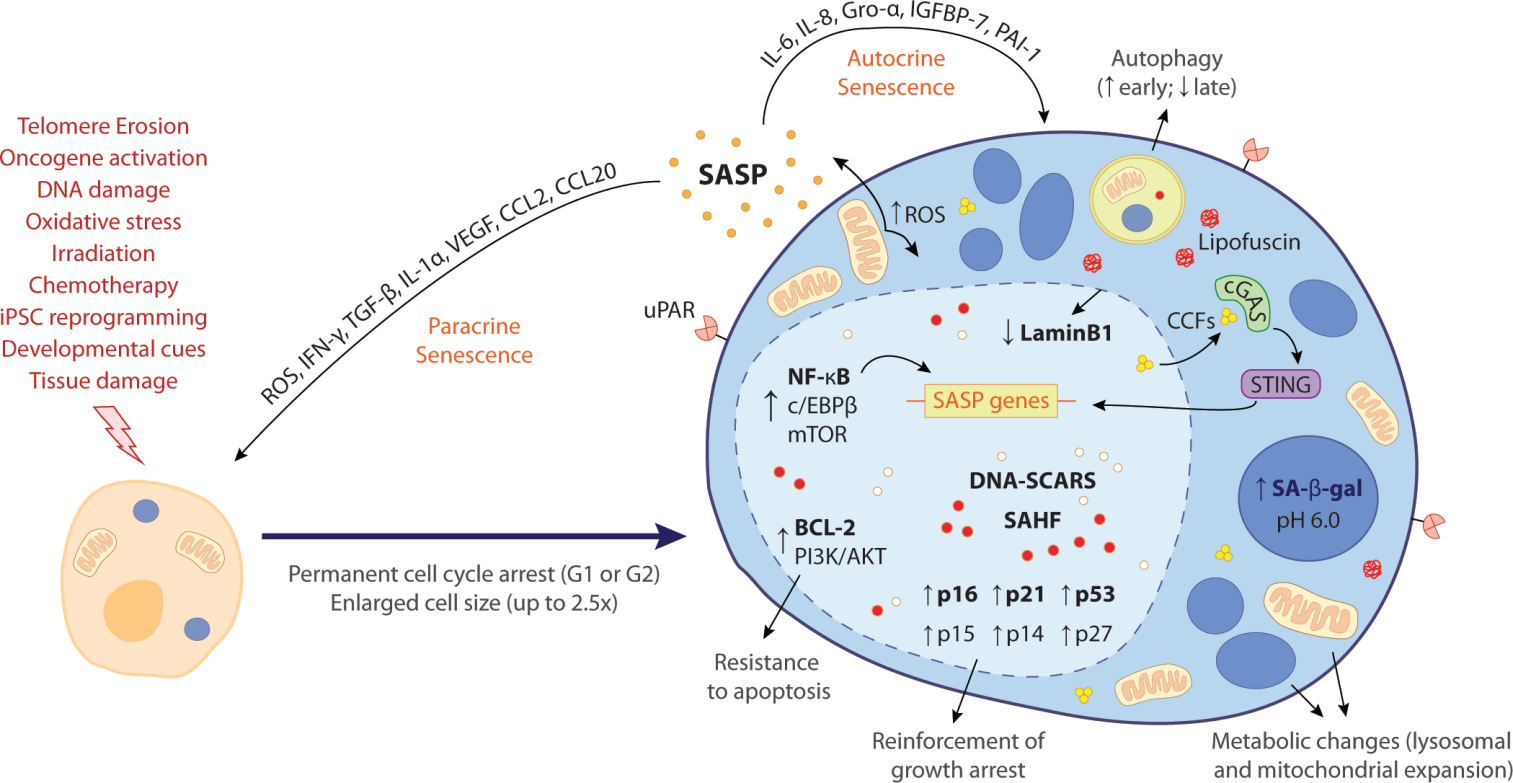
The hallmark features of cellular senescence. Cells may be induced to senesce by different stimuli, including telomere erosion, oncogene activation (oncogene-induced senescence [OIS]), DNA damage, oxidative stress, irradiation, chemotherapy, induced pluripotent stem cell (iPSC) reprogramming, developmental cues, and tissue damage. These stresses trigger the upregulation of key cell cycle inhibitors, namely p16^INK4a^, p21^CIP1^, p53, p15^INK4b^, p14^ARF^, and p27^KIP1^, leading to a permanent cell cycle arrest (either in G1 or G2 phase). BCL-2 and PI3K/AKT anti-apoptotic pathways also become upregulated, bestowing senescent cells (SCs) with resistance to apoptotic cues. Other phenotypic alterations include an enlarged cell size (up to 2.5 times the normal counterpart) and metabolic changes such as mitochondrial and lysosomal expansion, which increase the production of reactive oxygen species (ROS) as well as the activity of lysosomal senescence-associated β-galactosidase (SA-β-gal, detected at pH 6.0). ROS accumulation induces protein aggregates which crosslink with sugars and lipids and form insoluble lipofuscin aggresomes. Autophagy rate is increased during the early senescence programme but is highly compromised in later stages. SCs are characterized by a complex senescence-associated secretory phenotype (SASP) that comprises a plethora of different growth factors, cytokines, chemokines, proteases, and matrix components. Primary transcriptional activators of the SASP include nuclear factor kappa B (NF-κB), CCAAT/enhancer binding protein β (c/EBPβ), and mammalian target of rapamycin (mTOR). SASP factors, such as IL-6, IL-8, Gro-α, IGFBP-7, and PAI-1, reinforce the senescence programme in an autocrine manner, while others, like ROS, IFN-γ, TGF-β, IL-1α, VEGF, CCL2, and CCL20, induce paracrine senescence in neighbouring cells. Upon the initiation of the senescence programme, the chromatin suffers deep modifications towards the repression of proliferation-related genes and the stimulation of SASP-related genes. This results in the appearance of DNA segments with chromatin alterations reinforcing senescence (DNA-SCARS) as well as senescence-associated heterochromatic foci (SAHF). Chromatin alterations are accompanied by a downregulation of lamin B1, a major component of the nuclear lamina, compromising its integrity and leading to the extravasation of chromatin fragments into the cytosol. Cytosolic chromatin fragments (CCFs) are recognized by cyclic GMP-AMP synthase (cGAS) which, in turn, triggers the activation of stimulator of interferon genes (STING). The cGAS-STING pathway stimulates pro-inflammatory SASP responses through upregulation of NF-κB. Recent studies suggest that urokinase-type plasminogen activator receptor (uPAR), a cell-surface protein, is also broadly expressed during senescence. The biomarkers in bold represent the most common hallmark features of cellular senescence. None of these hallmarks is exclusively specific and their manifestation can diverge according to the nature of the senescence trigger, the cell/tissue type and time of the senescence programme.

Cellular senescence was formerly described by Hayflick and colleagues, after witnessing that normal diploid cells in culture had a limited number of cell divisions and entered a permanent cell cycle arrest (Hayflick and Moorhead, 1961). Hayflick observed that the nondividing cells remained viable for many weeks and suggested that this proliferative halt and finite lifetime in vitro represented aging at the cellular level (Hayflick, 1965). Several decades later, Hayflick’s proposal was corroborated by attributing this proliferation limit to a progressive telomere shortening after propagation of cells in culture, which is now known as replicative senescence (Harley et al., 1990). The link between replicative senescence and aging was further reinforced when an age-dependent accumulation of cells exhibiting senescence markers in vivo was revealed (Dimri et al., 1995). Two years later, a landmark study demonstrated that the expression of active mitogenic oncogenes (such as Ras) induced senescence in primary cells regardless of the age (Serrano et al., 1997). This process was coined oncogene-induced senescence (OIS) and introduced the concept of senescence working as a tumour-suppressive mechanism to prevent aberrant proliferation after an oncogenic stimulus. Since then, it has been established that cellular senescence can be induced by a wide range of different stress signals, such as oxidative stress (Chen et al., 1995), chemotherapy (Schmitt et al., 2002), induced-pluripotent stem (iPS) cell reprogramming (Krizhanovsky and Lowe, 2009), irradiation (Le et al., 2010), or cytokine treatment (Braumüller et al., 2013). It became then clear that senescence and aging are not synonymous and that senescent cells (SCs) can be triggered by several stressful insults independently of organismal age (Rodier and Campisi, 2011; Figure 1).

Importantly, SCs stopped being regarded as just undead or zombie cells that refuse to die. Though one might say they walk a fine line between life and death, it is now clear that SCs are much more than what was initially thought. In fact, they suffer numerous phenotypic changes and remain metabolically active cells with a complex secretory phenotype, through which they can modify the surrounding microenvironment (Muñoz-Espín and Serrano, 2014; Figure 1). Strikingly, SCs have recently emerged as beneficial players in various physiological processes, from embryonic development to cellular reprogramming and tissue injury responses such as wound healing and tissue repair (Rhinn et al., 2019).

### What truly defines a SC?

Cellular senescence has several biomarkers but none is absolutely specific (Figure 1). The cell cycle arrest is not exclusive of senescence and pRB and p53 are also involved in other forms of proliferative withdrawal (Rodier and Campisi, 2011). Even p16^INK4a^, one of the strongest senescence markers, might not be expressed in all SCs and is expressed in certain non-SCs (Hernandez-Segura et al., 2017; Sharpless and Sherr, 2015). The p53/p21^CIP1^ pathway does not always drive senescence and, in some tissues, cells that stain positively for senescence-associated β-galactosidase (SA-β-gal) lack the expression of p21^CIP1^ (Huang and Rivera-Pérez, 2014; Xu et al., 2019). In fact, despite its prominence, SA-β-gal is not a requirement of the senescent phenotype and it is possible to have SA-β-gal-negative SCs (e.g., those lacking GLB1) (Lee et al., 2006). In addition, false-positive SA-β-gal staining was also detected in macrophages (Hall et al., 2017). DNA segments with chromatin alterations reinforcing senescence (DNA-SCARS) and senescence-associated heterochromatic foci (SAHF) are stimulus-dependent and therefore cannot be regarded as a global feature of SCs (Di Micco et al., 2011; Kennedy et al., 2010; Rodier et al., 2011). The senescence-associated lipid profile seems to be variable depending on the trigger and mitochondrial dysfunction also portrays other cellular processes, so none of these are absolute biomarkers of senescence (Eisner et al., 2018; Quijano et al., 2012).

Even though senescence is traditionally associated with a transition into a proliferative arrest, it has become clear that terminally differentiated cells can develop a senescent phenotype. For instance, post-mitotic neurons in the cortex in both rodent and human aging brains were shown to exhibit several senescence features (Chinta et al., 2015; Jurk et al., 2012; Kang et al., 2015; Moreno-Blas et al., 2019; Walton and Andersen, 2019) and more recently, senescent neurons were also shown to be induced after spinal cord injury (Paramos-de-Carvalho et al., 2021).

Among the hallmarks of senescence, perhaps the most relevant one is the development of a complex secretory programme denominated as senescence-associated secretory phenotype (SASP), which was originally described by the Campisi group (Krtolica et al., 2001). Through the SASP, which comprises a plethora of different growth factors, chemokines, cytokines, extracellular matrix (ECM) components and proteases (Figure 1), SCs modulate the surrounding environment, exerting their pathophysiological effects (Le et al., 2010). Thus, cellular senescence has been implicated in a wide range of distinct biological activities, from aging and tumour progression or suppression to development, wound healing, and even regeneration (Rhinn et al., 2019; Rodier and Campisi, 2011).

The concept of paracrine senescence (senescence-induced senescence) is essential to understand the impact of the propagation of a senescence response in the tissue microenvironment. Continuous exposure to the SASP induces senescence in bystander normal cells, both in vitro and in vivo (Acosta et al., 2013; Nelson et al., 2012). Therefore, paracrine senescence has been shown to mediate the deleterious effects of SCs on tissue homeostasis, namely in promoting tumourigenesis and aging-related organ dysfunction, as well as in impairing regeneration (Campisi, 2005; Ferreira-Gonzalez et al., 2018; Gonzalez-Meljem et al., 2018).

Given the nature of the SASP, senescence signalling is often linked to inflammation. Remarkably, some SASP factors, such as colony stimulating factor 1 (CSF1), CCL2, and IL-8 recruit the immune system to promote self-clearance of SCs (Muñoz-Espín and Serrano, 2014). This seems to be critical to control the levels of SCs in a given setting and prevent chronic inflammatory responses.

It is clear that the manifestation of each senescence hallmark is context-dependent and varies according to factors such as the stress trigger, the cell or tissue type, and, last but not least, time from induction of the senescence programme (Calcinotto et al., 2019). Besides this huge heterogeneity, one must also account for the fact that non-SCs (even proliferative cells) may express some of the features normally associated with senescence. An example is the difficulty in often distinguishing whether secreted factors have a SASP origin or are molecules secreted by other non-SCs. This is why a multi-parametric approach is required in order to define a senescence signature. Data from transcriptome analysis and single-cell studies is proving to be a valuable tool in providing senescence-gene-expression signatures in several different conditions. For example, a proteomic atlas of SASP factors originating from senescent fibroblasts and epithelial cells submitted to multiple senescence inducers has been recently reported (Basisty et al., 2020). This type of comprehensive analysis is fundamental to define biomarker candidates with greater selectivity to specific pathological contexts, but information is still lacking and more studies are needed, especially in vivo. Meanwhile, a three-step multi-marker system has been proposed to identify SCs with more accuracy. This multi-marker workflow includes: (1) screening for senescence with SA-β-gal and/or lipofuscin accumulation; (2) co-staining with markers frequently present (p16^INK4a^, p21^CIP1^) or absent (proliferation markers, Lamin B1) in SCs; and (3) identification of factors predicted to be altered in specific senescence contexts (SASP, DNA damage, PI3K/FOXO/mTOR) (Gorgoulis et al., 2019).

### Senescence: the good, the bad, and the ugly

**Figure 2.**
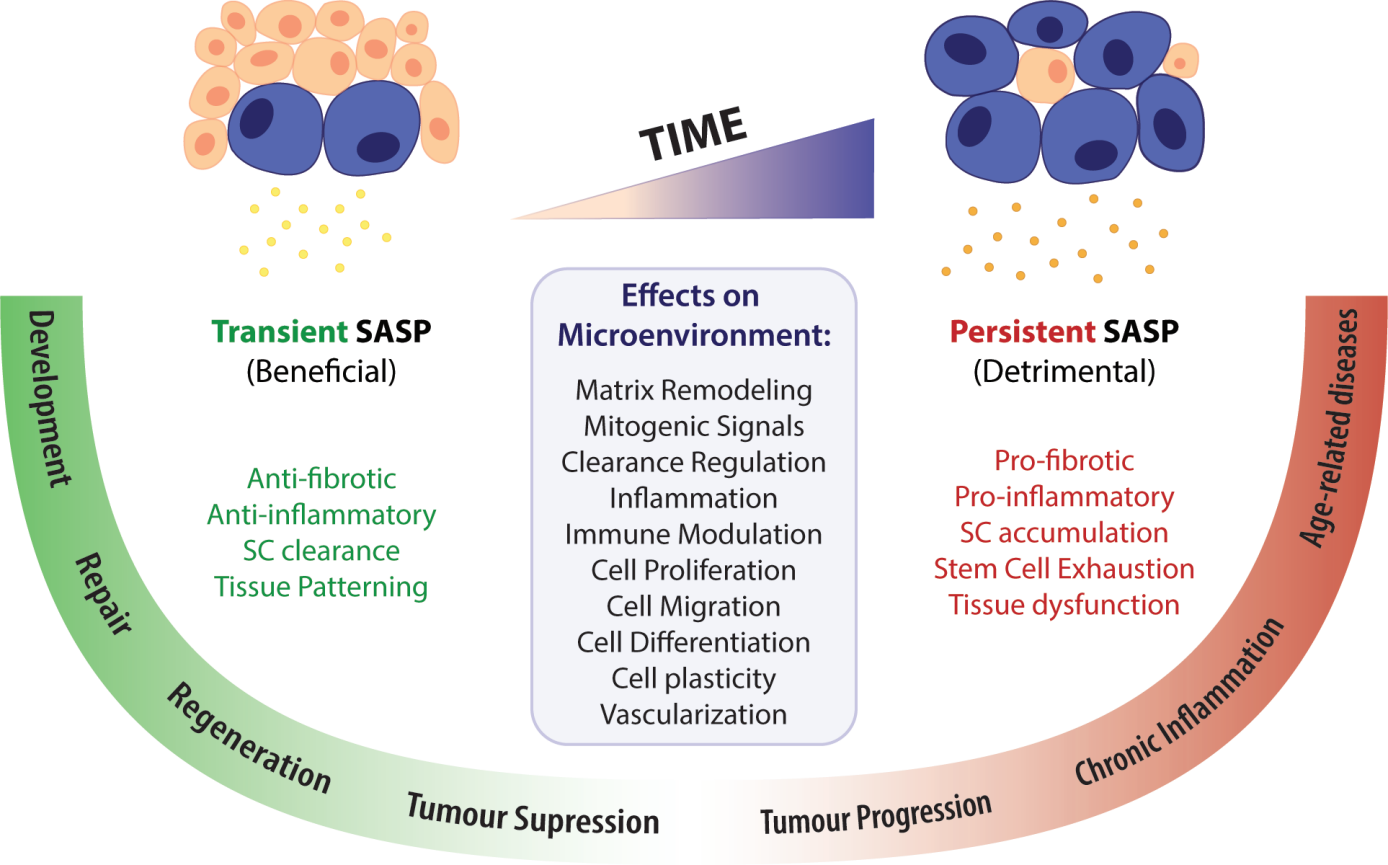
A time-gated model for the senescence-associated secretory phenotype (SASP)-mediated biological activities of cellular senescence. The SASP can have a wide range of effects in the surrounding microenvironment, including matrix remodelling, mitogenic signalling, clearance regulation, inflammation, immune modulation, cell proliferation, migration, differentiation and plasticity, as well as vascularization. Depending on the time duration of the senescence programme and the associated SASP response, effects can be beneficial or detrimental. Anti-fibrotic and anti-inflammatory effects are normally correlated to a transient SASP and favour tissue repair and regeneration. A short-term SASP also favours immune-mediated clearance of SCs in order to avoid their accumulation and persistence. Likewise, a transient senescence profile is fundamental for tissue patterning during development. In contrast, long-lasting SASP responses have detrimental pro-fibrotic and pro-inflammatory effects on the microenvironment. Therefore, the persistent accumulation of SCs leads to tissue dysfunction and is associated with chronic inflammation and a broad spectrum of aging-related diseases. Persistent senescence responses can also deplete stem cell progenitor pools, impairing the repair/regenerative capability of affected tissues. In turn, the role of the SASP in cancer is more ambiguous than the rest, as SCs can both promote tumour suppression and tumour progression/invasiveness. However, current knowledge suggests that the SASP suppresses tumour growth in early stages, while supplying pro-tumourigenic chronic inflammatory environments in later stages.

The discovery of additional cellular senescence features and biological roles provided a shifting view from a simple cell autonomous stress response to a dynamically active mobilization of environmental signalling cues with local or systemic repercussions for tissue function and, ultimately, organismal life. Some of these repercussions are beneficial while others are detrimental (Figure 2). In some contexts, these seemingly paradoxical roles are challenging to reconcile, justifying the apparent chaotic nature of this yet undeciphered phenomenon.

#### Aging

In zebrafish, rodents, primates, and humans, SCs are found in many tissues throughout life (Campisi, 2005; Dimri et al., 1995; Jeyapalan et al., 2007; Kishi, 2004). Though relatively rare in young organisms, they accumulate with age in several organs and tissues, such as the skin, heart, lung, spleen, kidney, and liver (Wang et al., 2009; Yang and Fogo, 2010). Aging entails a progressive loss of tissue functions that eventually leads to several chronic and age-related diseases (Campisi, 2013). So, a causal link between cellular senescence and aging seems quite plausible. In fact, senescence has been associated to a wide range of human age-associated pathologies, including cancer, fibrosis, cardiovascular diseases, type 2 diabetes, obesity, sarcopenia, osteoarthritis, osteoporosis, and neurological disorders (Calcinotto et al., 2019; van Deursen, 2014). Remarkably, the use of progeroid mouse models has provided evidence that ablation of p16^INK4a+^ cells improves many of these disease symptoms, reinforcing a detrimental role of SCs in aged tissues (Baker et al., 2011; Rhinn et al., 2019).

Some studies have also suggested that, with aging, senescence plays a role in the decline of regenerative capacity, namely by functionally depleting stem cell progenitor pools (Braun et al., 2012; Janzen et al., 2006). This was recently proposed during skeletal muscle regeneration in aged mice (Sousa-Victor et al., 2014). Through aging, geriatric muscle stem cells (MuSCs) suffer a quiescence-senescence switch triggered by an age-associated increase in p38MAPK activation and p16^INK4a^ expression, becoming unable to activate and expand upon injury. Importantly, inhibition of p38MAPK or p16^INK4a^ restored quiescence and regenerative functions in MuSCs (Cosgrove et al., 2014; Sousa-Victor et al., 2014). In the aging heart, cardiac progenitor cells (CPCs) progressively lose their regenerative capacity, as senescent CPCs render other healthy CPCs to senesce (Lewis-McDougall et al., 2019). It is still unclear if differentiated muscle cells develop senescent phenotypes, as they age, and consequently induce senescence in stem cell progenitor pools in a paracrine manner.

Similar to a ‘chicken or the egg’ paradox, one might wonder whether it is the accumulation of SCs that leads to aging or the opposite. The mechanisms behind SC accumulation with age and their influence on disease progression are still unclear. Some hypothesize that SCs develop ways to escape from immunosurveillance. In support of this, authors have shown that senescent dermal fibroblasts express atypical levels of MHC molecule HLA-E, induced by SASP-associated cytokines and regulated by p38MAPK (Pereira et al., 2019). HLA-E interacts with the inhibitory receptor NKG2A expressed by natural killer (NK) and highly differentiated CD8^+^ T cells, allowing senescent fibroblasts to evade immune clearance. Others theorize that, with age, the immune system becomes progressively compromised (immunosenescence) and cannot efficiently clear SCs as it should or that the generation rate of SCs is just too big to cope with (Furman et al., 2019). In any case, there is a very important notion to take from the relationship between senescence and aging: a persistent accumulation of SCs without a controlled clearance is detrimental for tissue function.

#### Cancer

Cellular senescence is a powerful barrier to tumourigenesis (Collado and Serrano, 2010). The first experimental evidence of OIS came from the overexpression of oncogenic Ras in human fibroblasts, resulting in upregulation of p53, p16^INK4a^, pRB, and permanent cell cycle arrest (Serrano et al., 1997). This can be bypassed by inactivation of p53 and p16^INK4a^ or co-expression of other oncogenes like c-MYC, E1A, or DRIL1 (Serrano et al., 1997). BRAF is another oncogene that promotes OIS by inducing p16^INK4a^ expression, in a process that requires the co-expression of IGFBP7 (Wajapeyee et al., 2008). The loss or inactivation of tumour suppressor genes, such as PTEN, also induces a senescence response (Chen et al., 2005). Loss of PTEN induces p53 through activation of mTOR and ARF-mediated inhibition of MDM2, but also p16^INK4a^ through upregulation of the transcription factor Ets2 (Ohtani et al., 2001). Contrarily to OIS, PTEN loss-induced senescence occurs in the absence of DNA damage response (DDR) but can also be bypassed by p53 inactivation (Ohtani et al., 2001).

The SASP of senescent tumour cells modulates the tumour microenvironment, which is also composed of non-senescent proliferating tumour cells, stromal cells, and infiltrating immune cells (Calcinotto et al., 2019). Thus, the SASP can act as another blocker of tumour growth by reinforcing autocrine senescence or inducing paracrine senescence in neighbouring tumour cells. For example, release of IL-1α by SCs spreads senescence to normal and tumour cells (Di Mitri and Alimonti, 2016). On the other hand, inhibition of IL-1α and IL-6 promotes OIS evasion (Di Mitri and Alimonti, 2016; Kuilman et al., 2008). Importantly, some SASP factors of tumour cells, such as TGF-β, VEGF, CCL2, and CCL20, also induce senescence in normal cells (Acosta et al., 2013). Highly relevant for tumour regression is the efficient removal of senescent tumour cells by immune cells that are recruited by the SASP itself (Vicente et al., 2016). Likewise, the immune response can then limit tumourigenesis by releasing factors, such as IFN-γ, TNF-α (tumour necrosis factor alpha), and TGF-β, in the tumour microenvironment and inducing senescence in tumour cells, generating a positive feedback loop (Braumüller et al., 2013; Calcinotto et al., 2019; Reimann et al., 2010). Yet, the SASP can also have immunosuppressive properties, attracting a high number of infiltrating myeloid-derived suppressor cells (MDSCs) that block the effect of other immune effector populations and inhibit the senescence response by releasing IL-1 receptor antagonist in the tumour microenvironment (Di Mitri et al., 2014).

Strikingly, the SASP of senescent tumour cells can also promote tumour progression, driving tumour vascularization and invasiveness through secretion of VEGF and a number of different matrix metalloproteinases (MMPs) (Coppé et al., 2006; Coppé et al., 2010). Several other SASP factors, such as Gro-α, basic fibroblast growth factor, and PAI-1, have been shown to stimulate malignant cell growth and cancer invasion. Secretion of IL-6 and IL-8 has been shown to promote both tumourigenesis and tumour suppression in different contexts (Rodier and Campisi, 2011). The SASP stands, therefore, as a double-edged sword, eliciting both antitumourigenic and tumour-promoting effects. SASP effects on cancer progression/suppression seem to rely not only on the context but also on the persistence of SCs through time. In line with this, a recent study has shown that telomerase-deficient zebrafish, who exhibit an accumulation of senescence similar to human aging, display increased systemic chronic inflammation through time, which potentiates cancer incidence and invasiveness (Lex et al., 2020). In fact, pre-cancer lesions developed into cancer at twice the rate if neighbouring tissues were senescent.

Chemotherapy drugs or ionizing radiation are used to induce senescence in cancer cells, in what is called therapy-induced senescence (TIS) (Calcinotto et al., 2019). The mechanisms underlying TIS are usually connected to the DDR. Presently, several TIS drugs are used for human cancer treatment, including Palbociclib, Doxorubicin, Bleomycin, Docetaxel, Cyclophosphamide, Etoposide, Vincristine, and Cisplatin (Ewald et al., 2010). Among all of them, the CDK4/6 inhibitor Palbociclib is currently considered the most relevant prosenescent compound in the clinic. However, in spite of its efficiency in blocking tumour cell proliferation, TIS must be used and monitored with attentive care. By also affecting normal cells, the senescence response might be so intense that the immune system fails to efficiently clear SCs from the tumour microenvironment. Indeed, the accumulation of SCs after TIS has been shown to promote tumour relapse with increased malignancy and premature aging features in human adults and children after chemotherapy (Marcoux et al., 2013; Ness et al., 2015). Furthermore, a recent study has demonstrated that, after TIS, some senescent tumour cells are able to escape from cell cycle blockade and acquire stemness properties with highly aggressive growth potential, which contradicts the terminal proliferative phenotype and has profound implications for the treatment outcome (Milanovic et al., 2018). This has been suggested as a form of tumour cell evasion from therapy, allowing their survival in a transient dormant state with potential to recover self-renewal capacity and lead to disease recurrence, normally with increased malignancy (Saleh et al., 2019). This led to a reassessment of treatment strategies, in view of taking advantage of the beneficial effects of senescence in blocking cancer cell proliferation but controlling subsequent detrimental outcomes due to its accumulation. As a result, current therapeutic strategies generally encompass a double approach, involving an initial pro-senescent step followed by an anti-senescent phase (Calcinotto et al., 2019). In summary, chemo/radiotherapy or senescence-inducing drugs can be combined with senolytic drugs that eliminate excess senescent tumour cells that are not efficiently cleared by the immune system. Additionally, modulation of the SASP or MDSC function (e.g. with antagonists of CXCR2, a receptor for several SASP cytokines) may be used to enhance the efficacy of pro-senescence therapies and promote senescence immune surveillance (Calcinotto et al., 2019; Toso et al., 2014).

The role of senescence in cancer rightfully epitomizes the good, the bad, and the ugly sides of this cellular phenomenon, by suppressing tumour growth in early stages, contributing to tumour development in later stage and eliciting tumour relapse and increased malignancy after arrest escape or chemotherapy. Importantly, with aging, this scenario becomes even more grim as SCs accumulate and supply pro-tumourigenic chronic inflammatory environments.

#### Development

The discovery of SCs throughout embryonic development was very exciting and brought relevant insights to better understand the physiological roles of senescence. So far, SCs have been found during development of mouse and human embryos (Chuprin et al., 2013; Muñoz-Espín et al., 2013; Storer et al., 2013), but also in naked mole rats (Zhao et al., 2018), birds (Gibaja et al., 2019; Nacher et al., 2006; Storer et al., 2013), amphibians (Davaapil et al., 2017; Villiard et al., 2017), and fish (Da Silva-Álvarez et al., 2020; Villiard et al., 2017) embryos and/or larvae. SCs in developing embryonic structures exhibit SA-β-gal activity and upregulation of p21^CIP1^, while expression of other senescence markers, like p53, p16^INK4a^, and DDR, is absent (Muñoz-Espín et al., 2013). This suggests a different senescence phenotype from that observed later in life and pinpoints the relevance of p21^CIP1^ in developmental senescence. Indeed, genetic disruption of p21^CIP1^ or senolytic treatment results in loss of senescence and patterning abnormalities in various structures (Davaapil et al., 2017; Gibaja et al., 2019; Muñoz-Espín et al., 2013; Storer et al., 2013). Interestingly, patterning defects are only observed transiently and are eventually compensated by other mechanisms, namely apoptosis (Muñoz-Espín et al., 2013; Storer et al., 2013). This suggests that SCs are intrinsic to development but not essential.

Overall, existing studies suggest that developmental senescence is not a damage-triggered event but rather a highly organized and programmed process with precise patterns in time and space, which may be orchestrated by other cues such as biophysical forces during morphogenesis. During development, SCs seem to contribute to tissue remodelling by controlling the balance of cell populations, fine-tuning of cell fate specification, morphogenetic signalling, and structural degeneration (the latter mediated by macrophage-dependent elimination).

#### Wound healing and tissue repair

A role of senescence in wound healing and tissue repair has been described in several organs, such as the liver (Krizhanovsky et al., 2008), the skin (Demaria et al., 2014; Jun and Lau, 2010), the heart (Meyer et al., 2016; Zhu et al., 2013), and the lung (Schafer et al., 2017; Table 1). In all cases, SCs seem to be closely associated with the levels of fibrotic tissue generated upon wound resolution, which in turn affect the outcome of the repair process (Baker et al., 2016; Yun, 2018). The deposition of ECM is critical for the maintenance of tissue integrity during wound healing but, if left unchecked, may lead to fibrosis and scarring. In the skin, CCN1, a matricellular protein expressed upon wound healing, elicits a senescence response in fibroblasts by triggering p53 and p16^INK4a^ via ERK and p38MAPK pathways (Jun and Lau, 2010). This response controls the proliferation of fibroblasts and ECM deposition, limiting the fibrotic scar and contributing to the healing process. Importantly, defects in CCN1 lead to fibrosis exacerbation (Jun and Lau, 2010). Also in the skin, in a more recent study using the p16-3MR transgenic model, Demaria and colleagues demonstrated that a transient accumulation of senescent fibroblasts and endothelial cells at the wound site induces myofibroblast differentiation through secretion of platelet growth factor AA (PDGF-AA) (Demaria et al., 2014). Elimination of p16^+^ cells resulted in delayed wound healing and increased fibrosis. Notably, topical administration of PDGF-AA reverted the delay in wound closure while maintaining the levels of fibrotic tissue, suggesting more SASP factors (likely MMPs) are involved in the healing process. In a mouse model of chronic liver damage, the administration of CCl_4_ induces fibrotic scarring and senescence in hepatic stellate cells (HSCs) also via a CCN1/p53/p16^INK4a^ pathway (Krizhanovsky et al., 2008). Senescent HSCs facilitate fibrotic resolution through secretion of MMPs. Finally, the cycle is completed by recruitment of NK cells to promote their own elimination. Moreover, p53;INK4A/ARF null mice displayed decreased numbers of senescent HSCs and extensive cirrhosis upon CCl_4_ treatment (Krizhanovsky et al., 2008). A time-controlled SASP-mediated clearance of SCs seems to be, in fact, a fundamental step for the outcome of the repair process. In a recent study using both human and mouse models of ischemic retinopathy, the SASP of senescent endothelial cells stimulated neutrophil recruitment and the extrusion of neutrophil extracellular traps, which both removed dysfunctional endothelial SCs and facilitated vascular pruning and repair (Binet et al., 2020).

**Table 1.** Senescence in tissue repair and regeneration.

Species	Model (tissue/type of injury)	Role (beneficial vs. detrimental)	Profile (transient vs. persistent)	Main findings	Biomarkers	SASP factors (identified)	Reference
Mouse	Cutaneous wound healing	**Beneficial**Anti-fibrotic.	N/E	- CCN1 → DNA damage/p53 activation + ROS-dependent p16^INK4a^/pRB activation → fibroblast senescence → ↑ expression of antifibrotic genes- CCN1 mutant mice → ↓ senescence → exacerbated fibrosis	SA-β-Gal, p16^INK4a^, p53	IL-6, IL-8, IL-11, MMP1, MMP3	Jun and Lau, 2010
Mouse	Cutaneous wound healing	**Beneficial**Anti-fibrotic.Promote differentiation.	Transient	- Fibroblast and endothelial cell senescence at the wound site → PDGF-AA secretion → myofibroblast differentiation- Elimination of p16^INK4a+^ cells → delayed wound healing + ↑ fibrosis	SA-β-Gal, p16^INK4a^, p21^CIP1^,↓ laminB1	PDGF-AA, VEGF, PAI-1, CCL5, and CCL2	Demaria et al., 2014
Mouse	Chronic liver damage	**Beneficial**Anti-fibrotic.Recruitment of immune system.	N/E	- CCl_4_ administration → activation of CCN1/p53/p16^INK4a^ pathway → hepatic stellate cell senescence → secretion of MMPs → fibrotic resolution → recruitment of NK cells to promote their own clearance- p53;INK4A/ARF null mice → ↓ senescence upon CCl_4_ treatment → ↑ cirrhosis	SA-β-Gal, p21^CIP1^, p53, HMGA1	MICA, IL-8, ULBP2, CD58,MMP1, MMP3	Krizhanovsky et al., 2008
Mouse	Heart infarction	**Beneficial**Anti-fibrotic.	Transient	- CCN1-dependent cardiac myofibroblast senescence → ↓ fibrosis → improved heart function- p53 deficiency → ↑ collagen deposition	SA-β-Gal, p16^INK4a^, p21^CIP1^, p53, p19^ARF^	MMP2, MMP9, IL-6, IL-11, CXCL1	Zhu et al., 2013
Mouse	Cardiac hypertrophy	**Beneficial**Anti-fibrotic.	N/E	- CCN1-dependent cardiac myofibroblast senescence → ↓ fibrosis → improved heart function- SC genetic ablation → ↑ fibrosis	SA-β-Gal, p16^INK4a^, p21^CIP1^,	N/E	Meyer et al., 2016;
Mouse	Cardiac ischaemia-reperfusion injury	**Detrimental**.Pro-fibrotic.Pro-inflammatory.	Persistent	- Oxidative stress → Cardiomyocyte and interstitial cell senescence → ↑ fibrosis and inflammation → impaired heart function- SC clearance → ↓ scar size and inflammation + ↑ myocardial vascularization → improved heart function	SA-β-Gal, p16^INK4a^, p21^CIP1^,	IP-10, TGF-β3, IL-6, IL-11, IL-16, CCL22, MIP-3β, CX3CL1	Dookun et al., 2020
Human/mouse	Ischemic retinopathy	**Beneficial**Recruitment of immune system.Pro-regenerative.	Transient	- Rapid proliferation of vascular cells → ↑ endothelial cell senescence → cytokine secretion+ neutrophil recruitment → formation of neutrophil extracellular traps (NETs) → removal of SCs → regression of pathological angiogenesis + regeneration of functional vessels- Inhibition of NETosis / neutrophil inactivation → impaired SC clearance → no regression of pathological angiogenesis	SA-β-Gal, PML	CXCL1, IL-1β	Binet et al., 2020
Mouse	Idiopathic pulmonary fibrosis (IPF)	**Detrimental**.Promote differentiation.Pro-fibrotic (indirectly).	Persistent	- Accumulation of senescent epithelial cells and fibroblasts → myofibroblast differentiation → ↑ fibrosis- SC elimination (genetic ablation and pharmacological) → improved pulmonary function and physical health	P16^INK4a^, p53, γH2AX	PAPPA, IGFBP2, IGFBP4, COL1A1, MMP10, MMP12, VCAM1, MCP1, PAI1, TNF-α	Schafer et al., 2017
Salamander	Limb amputation	N/E	Transient	- Turnover of SCs (unidentified) at injury site during regeneration	SA-β-Gal(in vivo)	N/E(in vivo)	Yun et al., 2015
Zebrafish/mouse (P1)	Heart injury	N/E	Transient	- Turnover of senescent cardiac fibroblasts during regeneration	SA-β-Gal, p53	N/E	Sarig et al., 2019
Zebrafish	Caudal fin amputation	**Beneficial**.	Transient	- Turnover of SCs (unidentified) at injury site during regeneration.SC removal → impaired regeneration	SA-β-Gal, p21^CIP1^	N/E	Da Silva‐Álvarez et al., 2019
Mouse	Acute muscle injury	**Beneficial**.Anti-inflammatory.Pro-regenerative.	Transient	- Senescence of fibro-adipogenic progenitors (FAPs) → cytokine secretion → anti-inflammatory environment → muscle regeneration	SA-β-Gal, γH2AX, p16^INK4a^, p53	TSG-6IL-33	Saito et al., 2020
Mouse	Biliary injury	**Detrimental**.Pro-fibrotic.Pro-inflammatory.Paracrine senescence.	Persistent	- Senescent cholangiocytes → TGF-β secretion → paracrine senescence of cholangiocytes and hepatocytes → ↑ fibrosis and inflammation → impaired liver regeneration- Inhibition of TGF-β-signaling → disruption of paracrine senescence → restoration of liver function	p16^INK4a^, p21^CIP1^, DCR2,γH2AX, p27, p53	TGF-β, IL1α	Ferreira-Gonzalez et al., 2018
Zebrafish	Spinal cord injury	N/E	Transient	- Turnover of senescent neurons at lesion periphery during regeneration	SA-β-Gal, p21^CIP1^,No BrdU	N/E	Paramos-de-Carvalho et al., 2021
Mouse	Spinal cord injury	**Detrimental**.Pro-inflammatory.Pro-fibrotic.	Persistent	- Continuous accumulation of senescent neurons at lesion periphery.- SC pharmacological depletion → ↑ myelin and axonal sparing + ↓ fibrosis and inflammation → improved motor and sensory functions	SA-β-Gal, p16^INK4a^,γH2AX	Amphiregulin, PDGF-BB, IGFBP-3, Serpin E1, IL-15, TNF- α, M-CSF, I-TAC, CCL11, ICAM-1, CCL20	Paramos-de-Carvalho et al., 2021

N/E - not explored.

The contrasting effects of transient vs. persistent senescence responses are further substantiated upon cardiac injury. In mouse models of cardiac hypertrophy and heart infarction, cardiac myofibroblasts enter senescence through a CCN1-dependent manner, reducing fibrosis in the short-term and improving heart function (Meyer et al., 2016; Zhu et al., 2013). However, after cardiac ischaemia-reperfusion injury, a long-term oxidative stress-induced senescence response in cardiomyocytes and interstitial cells promotes fibrosis and mediates a pro-inflammatory SASP, impairing heart function (Dookun et al., 2020). Similarly, in a mouse model of idiopathic pulmonary fibrosis (IPF), a chronic lung disease characterized by decreased lung function due to persistent scarring, senescent epithelial cells and fibroblasts accumulate continuously over time, inducing myofibroblast differentiation and exacerbating the fibrotic response (Schafer et al., 2017). In this case, the elimination of SCs improves the disease condition.

Together, these results suggest that timely and coordinated activation of SCs controls the early fibrotic response and is beneficial for wound repair. Yet, in the absence of proper clearance, persistent SCs accumulate and have a negative impact in tissue repair (Figure 2).

#### Regeneration

Though still a very understudied topic, recent findings suggest that SCs have indeed a part to play in regenerative processes (Table 1). In 2015, the team of Jeremy Brockes (UCL, London) reported a recurrent turnover of senescence during limb regeneration in the salamander (Yun et al., 2015). SCs were transiently induced near the amputation plane throughout regeneration and subsequently cleared by a macrophage-dependent immunosurveillance mechanism. This finding led to the obvious question of whether these transient SCs could be contributing to the regenerative response. Considering that salamanders regenerate limbs through dedifferentiation (Tanaka et al., 2016), this model constitutes a good system to study the impact of senescence on this particular process of cellular plasticity. However, due to the scarcity of tools to manipulate SCs in this model and assess their function, it is still unclear how senescence contributes to limb regeneration. One hypothesis is that SCs might stimulate dedifferentiation and/or create a more pro-regenerative microenvironment through the SASP (e.g., ECM remodelling, vascularization). Recruited immune cells, namely macrophages and NK cells, can also execute pro-regenerative functions (Godwin et al., 2013; Lucas et al., 2010; Rajagopalan and Long, 2012). Another possibility is that SCs work as a population balancing mechanism, controlling the proliferation of certain cell types during tissue re-growth and patterning, much like during development. Yet, it is possible that SCs are just a consequence of the local tissue damage cues from the injury and need to be removed, which then still raises the question of why not follow the apoptosis route instead. Whatever the case, current understanding of the functions of cell senescence tells us that these cells are likely modulating the surrounding microenvironment.

So far, very few studies have associated cellular senescence to regeneration, with most being accounted for during the past 5 years. In 2019, a transient burst of SA-β-gal^+^ cardiac fibroblasts was reported in zebrafish and neonatal mouse hearts after an injury (Sarig et al., 2019). Even though the effects of senescence on cardiac regeneration are yet to be determined, a study from 2015 demonstrated that telomerase-deficient zebrafish display an aberrant accumulation of SCs and fail to regenerate their hearts (Bednarek et al., 2015). Also in 2019, another study showed evidence of a transient accumulation of SCs after zebrafish caudal fin amputation (Da Silva‐Álvarez et al., 2019). In this case, tissue regeneration was impaired by removal of SCs with a senolytic drug, a type of drug that acts by inducing apoptosis in SCs (Zhu et al., 2015).

Yet, the association of senescence with regeneration phenomena goes beyond organisms with outstanding regenerative capabilities. In a mouse model of chronic inflammatory myopathy, senescence of fibro-adipogenic progenitors (FAPs) in response to exercise-induced muscle damage is necessary to promote functional recovery and muscle regeneration (Saito et al., 2020). In addition, the expression of p53 was found to be upregulated in the rat soleus muscle after a spinal cord injury (Graham et al., 2020). This upregulation disappears 3 months after injury, accompanied by a reduction in the expression of p16^INK4a^. Even though this study lacks the identification of some key senescence biomarkers, such as SA-β-gal and p21^CIP1^, it does seem to point to another transient turnover of SCs in tissues with regenerative capacity.

In a mouse model of biliary injury, TGF-β secretion-mediated paracrine senescence of cholangiocytes and hepatocytes was shown to have deleterious repercussions in the surrounding microenvironment and impair liver regeneration (Ferreira-Gonzalez et al., 2018). The inhibition of TGF-β signalling disrupted the continuous propagation of senescence, reduced collagen deposition, and restored liver function. This work attests the detrimental impact of a persistent SASP exposure in regenerative paradigms.

In our lab, we have recently provided evidence that there are distinct senescence responses induced after a spinal cord injury between two animal models with different regenerative capabilities (Paramos-de-Carvalho et al., 2021). In this study, the regenerating zebrafish exhibits a transient accumulation of SCs at the lesion periphery, which are then cleared out. In contrast, in the injured mice SCs persistently accumulate over time and are not eliminated. Targeting of SCs in the mouse spinal cord in order to prevent their accumulation after injury resulted in improved motor, sensory, and bladder functions, supported by beneficial effects on myelin preservation, axonal growth, fibrotic resolution, and inflammation. These effects were accompanied by a reduced secretion of pro-fibrotic and pro-inflammatory factors in the injury microenvironment (Table 1). This work highlights the role of SCs in modulating the spinal cord injury microenvironment, which is permissive for regeneration in the zebrafish but is inhibitory in mammals.

Transversal to all these reports is the timely elimination of SCs, which suggests that their persistence could eventually turn detrimental for the regenerative response. Current knowledge seems to support this hypothesis, given the outcome of transient vs. persistent SASP exposure in pathological conditions or aging.

#### Reprogramming

Recent studies have uncovered another unexpected facet of senescence: a role in cellular reprogramming. This facet became even more interesting when it was discovered that senescence has opposite effects in vitro and in vivo reprogramming. In vitro, the expression of the four Yamanaka factors (OCT3/4, SOX2, KLF4 and c-MYC, OSKM) activates senescence markers in targeting cells, such as SA-β-gal, p16^INK4a^, p21^CIP1^, and SAHF (Banito et al., 2009). This seemingly intrinsic barrier to reprogramming probably explains why the iPS cell generation efficiency is so low (≈0.02%) (Takahashi et al., 2007; Takahashi and Yamanaka, 2006). In fact, silencing of senescence-associated genes like p53, CIP1, and INK4a has been shown to increase iPS cell generation rate from mouse and human fibroblasts (Banito et al., 2009; Hong et al., 2009; Li et al., 2009). Strikingly, cellular senescence exerts a contrary effect on in vivo reprogramming. Such has been demonstrated in mice engineered to transiently express the four OSKM factors in an inducible fashion (i4F). i4F wild-type mice exhibited co-expression of SA-β-gal and NANOG, a pluripotency marker (Mosteiro et al., 2016). Without INK4a/ARF, this co-expression was lost. Remarkably, in mouse models of lung and muscle damage in which senescence is induced at the lesion sites, the efficiency of reprogramming of i4F mice was increased (Chiche et al., 2017; Mosteiro et al., 2016). This efficiency was also enhanced in conditions that elicit senescence activation, including Palbociclib treatment, irradiation, and even aging. On the other hand, reprogramming was decreased after p16^INK4a^ deletion or treatment with the senolytic drug ABT-263 (Chiche et al., 2017; Mosteiro et al., 2016). This senescence-induced favourable environment for reprogramming was found to be mediated by secretion of the SASP factor IL-6 that activates the JAK/STAT target PIM1 to promote cellular plasticity, which can be reverted by treatment with IL-6 antibodies (Chiche et al., 2017; Mosteiro et al., 2016). In another study, Ras-mediated OIS in the skin and liver was shown to induce the expression of stemness markers, such as CD34, Lgr6, and Nestin, in senescent keratinocytes (Ritschka et al., 2017). This was also demonstrated to be SASP-dependent, as it was abolished with nuclear factor kappa B (NF-κB) inhibition. However, the expression of stemness markers was overcome by senescence features upon a longer (6 days) exposure to the OIS-driven SASP, which was proposed as a cell-intrinsic anti-tumourigenic response to counteract stemness. It is interesting to speculate that there is a risk that a short exposure to an oncogenic trigger can initiate a premature senescent response entailing a stemness profile with aberrant plasticity and high tumour-initiating capability. In any case, the relationship between senescence and reprogramming seems to be defined by the time of exposure to the SASP.

### Targeting cellular senescence

The role of senescence in diverse pathological settings has elevated SCs to a hot target in a wide range of therapeutic approaches for injured or aged tissues (Paez-Ribes et al., 2019). Despite the proposition that removing SCs might extend lifespan, it becomes imperative to consider the potential drawbacks of removing SCs from certain tissues. In fact, a recent study has proven that senescent liver sinusoid endothelial cells have important structural and functional roles in the aging organism and cannot be replaced after removal (Grosse et al., 2020). Consequently, their acute elimination promoted liver and perivascular tissue fibrosis, as well as health deterioration. Nonetheless, in aging-related scenarios, targeting senescence has become considered targeting aging itself. Thus, the last few years have witnessed a boom in the development of senescence-targeted strategic tools with translational impact. Given the lack of a specific biomarker that could be used for specific targeting, current senescence-directed therapeutic approaches rely on three main strategies: (1) interference with important senescence pathways (e.g., pro-survival); (2) manipulation of the SASP; and (3) immune system activation (Paez-Ribes et al., 2019).

The work of Zhu et al., 2015, established the senolytics as a class of drugs that induce apoptosis in SCs by interfering with pathways that are crucial for the maintenance of the senescence phenotype. Transcriptomic analysis revealed the upregulation of pro-survival genes in SCs, namely those involving the anti-apoptotic PI3K/AKT pathway and BCL-2 family proteins, as compared to normal cells (Zhu et al., 2015), and identified drugs that can counteract the pro-survival genes and cause apoptosis. Senolytic approaches are currently the leading strategy to promote SC elimination in vivo (Paez-Ribes et al., 2019). Senolytic agents such as Dasatinib, Quercetin, and Navitoclax (also known as ABT-263) are being tested in several clinical trials for the treatment of specific types of senescence/age-associated disorders and cancer (Paez-Ribes et al., 2019). However, concerns with specificity and unwanted side effects may prove limiting factors for therapeutic development, hindering their translation into clinical interventions.

A specific modulation of the detrimental effects of the SASP without compromising the cell cycle arrest can be therapeutically advantageous. Of course, this requires a deep characterization of the SASP nature in each particular setting. In view of this, a number of molecules and antibodies aiming at interfering with transcriptional activators of the SASP (such as NF-κB, p38MAPK, and C/EBPβ) or neutralizing specific factors have already been developed (Paez-Ribes et al., 2019). Such compounds are called senostatics. Similarly to senolytics, there are essential aspects to consider when designing senostatics. First, all SASP transcriptional regulators also have non-senescence-associated functions and, thus, their targeting might generate undesirable side effects (Kang, 2019). Second, the SASP is very heterogeneous and the beneficial vs. the detrimental role of certain SASP factors seem to be context- or at least time-dependent. Therefore, a generalized inhibition of the SASP may prove disadvantageous.

The third strategy to target SCs consists on sensitizing immune cells to promote their clearance, a concept that may acquire particular relevance considering that the accumulation of SCs in aged tissues is thought to partially result from the development of escape mechanisms from immunosurveillance or a generally declining immune system (immunosenescence) (Furman et al., 2019). In vitro studies have reported evidence for NK and CD4^+^ T cell sensitization towards certain types of SCs upon administration of anti-DPP4 (a surface peptidase) and anti-Vimentin (membrane-bound protein) antibodies, respectively (Frescas et al., 2017; Kim et al., 2017). Recently, a ground-breaking study reported the senolytic potential of chimeric antigen receptor (CAR) T cells that target urokinase-type plasminogen activator receptor (uPAR), a cell-surface protein that was found to be broadly expressed during senescence (Figure 1; Amor et al., 2020). uPAR-specific CAR T cells were shown to efficiently eliminate SCs in different in vitro and in vivo contexts, unveiling a promising therapeutic strategy for senescence-associated pathologies.

A major advantage of adopting an immune sensitization strategy towards the use of senolytics and senostatics, which may systemically target pathways or factors that are not exclusive of senescence, is the efficiency in redirecting immune cells to target SCs with high specificity. Specificity is particularly relevant since we know that SCs can upregulate different markers depending on the context in which the senescence programme is triggered. For example, a recent study has established an association between the upregulation of CD9 (a membrane protein known to regulate cell adhesion and migration) in endothelial cells and atherosclerotic plaque formation (Cho et al., 2020). CD9 regulated cellular senescence through a phosphatidylinositide 3 kinase-AKT-mTOR-p53 signalling pathway and its expression increased in arterial tissues with age, contributing to the pathogenesis of atherosclerosis. In this context, specifically targeting CD9 may represent an ideal strategy for prevention and treatment of vascular aging.

From a therapeutic point of view, there is an urgent need of reliable tools for detection and targeting of different types of SCs in vivo. Nowadays, the development of novel fluorescent probes and the recent advances in nanotechnology are providing promising tools (e.g., theranostics) with serious translational potential for diagnosis and targeted treatment of senescence-associated disorders (Paez-Ribes et al., 2019). However, these technologies need further exploration before clinical implementation.

Recently, it has been proposed that cellular senescence, contrarily to what was long thought, can be reverted. In 2018, Kornicka and colleagues demonstrated that a combination of 5‐Azacytydine and Resveratrol reversed the senescent phenotype of adipose stem cells through modulation of mitochondrial dynamics and autophagy (Kornicka et al., 2019). Upon this combinatorial treatment, adipose stem cells exhibited increased proliferation rate, decreased SA-β-gal activity, and lower reactive oxygen species (ROS) accumulation. Similar results were found in replicative senescent retinal pigment epithelial (RPE) cells co-cultured with embryonic stem cells (Wang et al., 2020). In this case, senescent RPE cells displayed increased proliferative capacity re-entering the S and G2/M cycle phases, along with the downregulation of several senescence biomarkers, such as SA-β-gal, p53, p21^CIP1^, p16^INK4a^. Though the mechanisms by which the senescent state can be reverted are still unclear, it has been shown that 3-phosphoinositide-dependent protein kinase 1 (PDK1) inhibition suppresses NF-κB and mTOR signalling and abolishes senescence hallmarks in senescent human dermal fibroblasts, restoring their quiescent state (An et al., 2020). The development of ‘senoreverters’ is thus rising as a promising alternative strategy to target SCs.

### Hanging questions and considerations

While much progress has been achieved in understanding the biological roles of senescence, its apparently chaotic nature may yet reveal deeper complexities. How can we reconcile such a heterogeneous and puzzling phenomenon? Are there common fingerprints across different settings? In time, single-cell transcriptional analysis combined with machine learning tools will yield invaluable information on context-specific senescent signatures and patterns.

Cellular senescence seems, fundamentally, a response to stress. It is becoming increasingly evident that SCs can be found in virtually any tissue. And, even though considerable knowledge was acquired on the possible different triggers of the senescence programme, it is still unclear how SCs are induced in vivo. Once this programme starts, how extensively does it modify the cellular phenotype in time? In other words, is a senescent fibroblast still a fibroblast? The same question will apply to every cell type. Importantly, the senescence programme entails deep epigenetic alterations which permit a tight regulation of transcriptional activities that is essential to its timely execution. This network was found to be orchestrated by activator protein 1 (AP-1), who ‘imprints’ the senescence enhancer landscape that drives the transcriptional programme of SCs and determines their fate (Martínez-Zamudio et al., 2020). Upon a stress, what factors determine whether a cell undergoes senescence and not apoptosis? It has been suggested that the balance between senescence and apoptosis upon genotoxic stress is regulated by high mobility group box‐1 (HMGB1) proteins (Lee et al., 2019). In addition, a crosstalk between the telomere shortening/p53 and AKT/FoxO signalling pathways has been proposed to regulate an apoptosis-to-senescence switch during aging (El Maï et al., 2020).

It came as a surprise that senescence has a role to play in organismal life since very early on and may have originally arisen as a developmental mechanism. In fact, organs of regenerating animals, such as the zebrafish, often display senescence features during their developmental stages (Da Silva-Álvarez et al., 2020), suggesting a link between regeneration and the recapitulation of developmental senescence. However, in non-regenerating scenarios, SCs exhibit disparate behaviours that lack a tight regulation like the one seen during embryogenesis.

The recent findings on the role of SCs in tissue injury and regenerative responses are quite exciting. Though their functions remain mostly undeciphered, SCs have been shown to facilitate fibrotic resolution and also suggested to modulate cell plasticity during injury responses. These beneficial roles are primarily attributed to the SASP and are considered therapeutically relevant for pro-regenerative interventions. However, it is particularly pertinent to consider that many SASP factors are also known tissue damage signals secreted by non-SCs. Without a transcriptional analysis at the single-cell level, it will be hard to distinguish between them.

After all, are SCs good Samaritans or camouflaged villains? In this perspective, the reconciling factor seems to be ‘time’. The notion that the functions of SCs are orchestrated by temporally regulated mechanisms is probably the most coherent senescence concept so far. While the beneficial roles of senescence all share a transient profile, the deleterious functions of SCs are associated to their lingering persistence, namely the chronic exposure to their SASP (Figure 2; Rhinn et al., 2019).

Targeting SCs is, for all the aforementioned reasons, currently a trending topic, so much so that several biotechnology companies are investing in the development of SC-targeted therapies with translational relevance. Yet, the lack of a specific senescence biomarker remains a limiting factor for efficient translation. Moreover, there are important concerns regarding the clinical application of existing approaches. First, when is the right time to target senescence? Senescent profiles are highly dynamic and heterogeneous. Therefore, the timeframe of each senescence response should be accurately assessed in each particular setting in order to define an optimal therapeutic time-window. Second, how does the evolving senescence programme affect the feasibility of a senotherapy? It is known that each senolytic, senostatic, or immunosensitization approach targets a specific hallmark of senescence. In the case of the first two, it is usually an upregulated signalling pathway which is key to sustaining the senescent phenotype and/or cell viability, while in the case of the latter can be any expressed marker deemed specific for a given SC type. However, it has become clear that the expression level of these hallmarks can significantly vary along the maturation of the senescence programme/response. These variations can thus determine the efficacy and success of distinct senotherapies in each particular point in time. Only a full time-wise phenotypic characterization can guarantee the optimal adequacy and effectiveness of a designed senotherapy. Third, SCs play important physiological roles in some specific contexts (e.g., wound healing) and, therefore, their general targeting must be carefully evaluated. This may be further complicated by the existence of different SC populations within the same context, as was recently demonstrated in the mouse liver where two distinct populations of p16^INK4a+^ and p21^CIP1+^ SCs have been shown to respectively promote and impair the regenerative capacity of the organ (Ritschka et al., 2020). Therefore, the selectivity of existing senolytics/senostatics must be improved and their delivery must be planned according to space and time. Nanoparticles represent a promising tool to specifically target pathological SCs in certain tissues, while diminishing drug-associated toxicity side effects, but this technology still needs further characterization and development. Ideally, targeting the SASP should aim at suppressing its deleterious effects while keeping or harnessing its beneficial roles. Yet, this will only be possible after an in-depth functional characterization of each SASP component in each different context. Another concern is that, given the multifaceted nature of SCs and the differences between humans and mice, existing pre-clinical studies might not reflect the complex microenvironment of diseased tissues from human aging-related disorders. Therefore, when possible, SC characterization and senotherapy validation should be performed in human samples ex vivo obtained from different contexts. In the future, this will likely be fundamental to generate personalized anti-senescent strategies.

A serious question to consider is whether, in the end, we really want to eliminate cells that were once a fundamental and integral part of our organismal homeostasis. This may be critical in tissues without the ability to regenerate or repopulate lost cells, such as the central nervous system. In certain scenarios, a revolutionary strategy would reside in being able to efficiently revert SCs to their original state. On this matter, the development of ‘senoreverters’ may emerge as a valuable targeting strategy for cellular senescence. However, we have seen that in certain contexts, like in cancer, the reversion of a pre-senescent state may result in highly aggressive growth behaviours. Thus, the possible repercussions of using tools to revert the senescent state should be prudently examined. While such tools are yet to be refined, the *pros* and *cons* of targeting SCs should always be cautiously weighed in the balance. Beyond the *how*, finding the right *when* may prove to be the key for the success of a senotherapy. It is becoming increasingly clear that, in senescence, *time* is of the essence.
